# Association of circulating omega 3, 6 and 9 fatty acids with gestational diabetes mellitus: a systematic review

**DOI:** 10.1186/s12902-021-00783-w

**Published:** 2021-06-15

**Authors:** Shaghayegh Hosseinkhani, Hojat Dehghanbanadaki, Hossein Aazami, Parvin Pasalar, Mojgan Asadi, Farideh Razi

**Affiliations:** 1 Department of Clinical Biochemistry, School of Medicine, Tehran University of Medical Sciences, Tehran, Iran; 2 Metabolomics and Genomics Research Center, Endocrinology and Metabolism Molecular-Cellular Sciences Institute, Tehran University of Medical Sciences, Tehran, Iran; 3 Endocrinology and Metabolism Research Center, Endocrinology and Metabolism Clinical Sciences Institute, Tehran University of Medical Sciences, Tehran, Iran; 4 Diabetes Research Center, Endocrinology and Metabolism Clinical Sciences Institute, Tehran University of Medical Sciences, Tehran, Iran

**Keywords:** Omega fatty acids, Gestational diabetes mellitus, GDM, Maternal lipids, Systematic review

## Abstract

**Background:**

Gestational diabetes mellitus (GDM) is associated with increased risks of disease for mother and child during pregnancy and after that. Early diagnosis of GDM would promote both maternal and fetal health. Metabolomics can simplify and develop our understanding of the etiology, manifestation, or pathophysiology of the disease. This systematic review investigates the association of circulating omega 3, 6, and 9 fatty acids with GDM.

**Methods:**

We conducted a systematic search of PubMed, Scopus, Web of Science, and EMBASE databases up to May 8, 2020, using the key term combinations of all types of omega fatty acids with gestational diabetes mellitus. Additional articles were identified through searching the reference lists of included studies.

**Results:**

This systematic review included 15 articles. Five were cohort studies, four included nested case-control studies and four were case-control studies. The results of this study demonstrate an increasing trend in the amount of oleic acid and palmitoleic acid in the second trimester and an increase in decosahexanoic acid in the third trimester of GDM mothers. The changes in other fatty acids of interest are either not significant or if significant, their results are inconsistent with the other existing articles.

**Conclusions:**

Omega fatty acids, as potential biomarkers, are considered to be associated with GDM risk and thus provide useful information regarding the prevention and early diagnosis of GDM. Moreover, existing metabolomic studies on GDM are shown to provide conflicting results about metabolite profile characteristics. This systematic review was registered at PROSPERO (www.crd.york.ac.uk/PROSPERO) as CRD42020196122.

## Background

Gestational diabetes mellitus (GDM) is a hyperglycemia state that develops or is first recognized during pregnancy. Development of GDM increases the risk for adverse outcomes such as hypoglycemia, respiratory distress syndrome, and macrosomia in the newborns, as well as increased risk of cesarean section and development postpartum type 2 diabetes (T2D) in mothers [1]. Maternal obesity is the most important risk factor for gestational diabetes. Also, genetic factors, dietary habits, environmental factors, and lower knowledge and education about healthy behavior have a potential role in the development of gestational diabetes [2, 3]. It is also crucial to note that, due to an increasing prevalence of obesity and diabetes mellitus worldwide, the number of pregnant women with GDM is rising.

Altered maternal lipid metabolism in pregnancy with a moderate increase of lipids early in pregnancy and a significant increase of lipids later in pregnancy is also prevalent [2]. Studies support primary, and potential effects of prenatal long-chain omega 3 polyunsaturated fatty acid (LC-n3 PUFAs) amounts on offspring anthropometry and body composition measures [4]. Moreover, the fetal PUFA concentrations and composition essentially depend on the maternal metabolic status because fetal PUFA synthesis is limited and they are transferred to the fetus through the placenta [5]. Maternal fatty acid and lipid metabolism change during pregnancy to facilitate fetal growth and development. Adequate amounts of fat is required during pregnancy as both deficiency and excess consumption can have negative effects on fetal as well as maternal health [6]. Excessive consumption of fatty foods or imbalance in the consumption of different types of fats, especially omega 3 and omega 6 fatty acids as essential fatty acids and saturated fatty acids, plus changes in fatty acid metabolism during pregnancy can potentially lead to an increased risk for GDM by developing insulin resistance and increasing inflammatory factors. Maternal fatty acid metabolism changes during pregnancy, especially in regards with PUFAs which are reflected in the blood [7]. This matter is considered to be due to insulin resistance in adipose tissues and the release of excess fat into the bloodstream [7]. We can get a good interpretation of fatty acid metabolism changes during pregnancy and its relevant complications such as GDM by assessing the concentrations of omega fatty acids during pregnancy is capable of providing us with an excellent interpretation of changes in fatty acid metabolism and other relevant complications, and also gives us the chance to use them as predictors or early diagnostic biomarkers [8].

Metabolomics as a downstream product of gene expression can facilitate our understanding of the etiology, manifestation, or pathophysiology of this disease. Compared to biomarkers that are clinically common and diagnostic approaches that are used, metabolomics also provides proper sensitivity and accuracy. Metabolomics has a promising vision for identifying and measuring considerable metabolic characteristics which lead to pathological conditions like GDM. Studies have employed nuclear magnetic resonance (NMR), targeted or complementary non-targeted gas/liquid chromatography-mass spectrometry (GC/ LC-MS) based assays, etc. to evaluate lipid profiles in GDM [9]. Lipidomics studies have led to advances in the structural recognition, classification, and naming of lipids. However, due to the structural and functional complexities of lipids as well as the diversity of their abundance in biological samples, more comprehensive studies with strong methodologies are needed to better understand these compounds [10].

There is a major debate in the literature as to whether lipid patterns differ in women with GDM early in pregnancy and if these early patterns are potential markers of preexisting insulin resistance [2]. So, in this study, we intend to systematically investigate the literature to evaluate the omega-3,6 and 9 fatty acid levels in women with GDM, compared to healthy pregnant women based on metabolomic analytical techniques.

## Methods

This systematic review is in accordance with the preferred reporting items for the systematic reviews and meta-analysis (PRISMA) statement.

1. Protocol and registration
  The systematic review was registered at PROSPERO (www.crd.york.ac.uk/PROSPERO) as CRD42020196122.
2. Eligibility criteria

For the eligible studies, inclusion criteria included: (1) case-control or cohort studies design, (2) comparing the concentration levels of omega 3, 6, and 9 fatty acids in maternal blood, serum, or plasma, (3) participants included pregnant women classified as having GDM and controls (healthy pregnant women with normal glucose tolerance), (4) metabolomic techniques such as GC-MS or NMR spectroscopy were used to measure metabolite profile; and (5) published in English with the full-text articles available.

Exclusion criteria for the articles included: (1) duplicated publications, (2) animal studies, (3) pre-pregnancy diabetes, (4) letters, meta-analyses, reviews, expert opinions, case series, case reports, also articles that did not meet the inclusion criteria were excluded.

3) Information sources & Search

A comprehensive literature search was carried out in the PubMed, Scopus, Web of Science, and EMBASE databases to obtain relevant studies and the cut-off date was up to May 8, 2020. Also, the reference list of all included articles was analyzed carefully to recognize additional articles.

We used the following synonyms obtained from PubMed Mesh terms: “hypogeic acid” OR “Oleic Acid” OR “elaidic acid” OR “gondoic acid” OR “mead acid” OR “erucic acid” OR “nervonic acid” OR “ximenic acid” OR “Hexadecatrienoic acid OR “alpha-linolenic acid” OR “stearidonic acid” OR “Eicosatetraenoic acid” OR “Eicosapentaenoic Acid” OR “heneicosapentaenoic acid” OR “Clupanodonic acid” OR “Docosahexaenoic Acids” OR “Tetracosahexaenoic acid “OR “Tetracosapentaenoic acid” OR “Linoleic Acid” OR “gamma linolenic acid” OR “Calendic acid” OR “Eicosadienoic acid” OR “Eicosatrienoic Acid” OR “Arachidonic Acid” OR “Docosadienoic acid” OR “Adrenic acid” OR “Tetracosatetraenoic acid” OR “tetracosapentaenoic acid” OR AND (“Diabetes, Gestational“[Mesh] OR “Pregnancy-Induced Diabetes” OR “Pregnancy Induced Diabetes” OR “gestational diabetes”)

4) Study selection & Data collection process & Data items

Study selection and data collection from eligible studies were independently evaluated by two investigators. A third person resolved the differences until all reached a set of similar statements. Data on population characteristics and metabolomic techniques and study results were extracted from all eligible studies. Due to the limited number of GDM and metabolic studies, methodological heterogeneity, and considerable differences in the characteristics of the study populations, a quantitative meta-analysis could not be done.

## Results

### Study selection

**Fig. 1:**
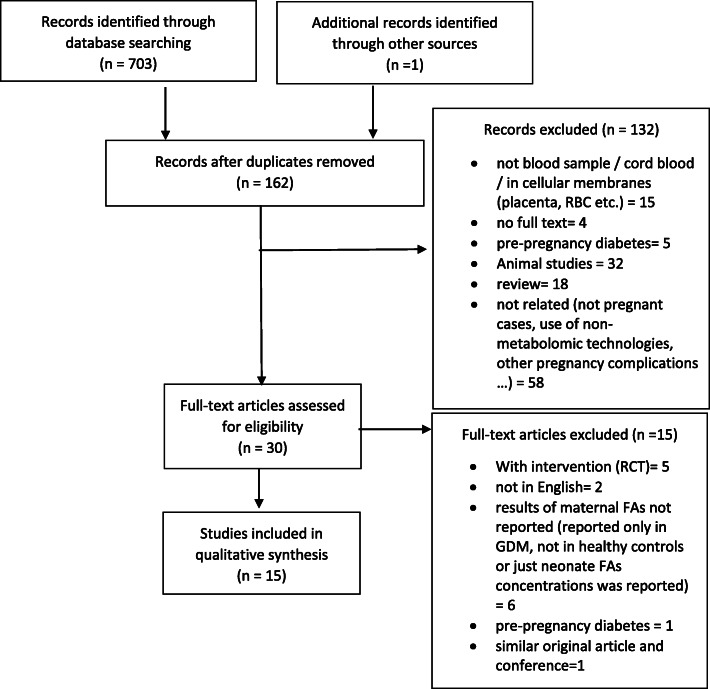
The algorithm of systematic review study selection

We identified 703 records from Scopus (*n* = 228), Web of Science (*n* = 117), PubMed (*n* = 90) and EMBASE (*n* = 268) and 1 article identified through manually searching bibliographies. After the elimination of duplicates (*n* = 542), 162 records remained. The selection algorithm for the 15 studies that met the inclusion/exclusion criteria is detailed in Fig. 1.

### Study characteristic

Included articles were 10 original articles that we had access to their full text [11–20] and 5 conference abstracts [21–25]. Five were cohort studies [11, 12, 21, 23, 25], four were nested case-control studies [13, 14, 18, 20] and four were case-control studies [15–17, 19].

The studies used various GDM diagnostic criteria as follows: 5 studies used Carpenter–Coustan criteria [12, 14, 16, 18, 20], 3 studies referenced the International Association of Diabetes and Pregnancy Study Groups (IADPSG) criteria [11, 13, 15], 1 study referenced the National Diabetes Data Group (NDDG) criteria [19], 1 study specified using O’Sullivan and Mahan criteria [17], and 5 studies did not specify the GDM diagnostic criteria used [21–25]. Briefly, IADPSG (and WHO) suggest a one-step 2 h approach with oral 75 g glucose loading. If one result was found to be more than the glucose cut-off value (fasting: 92 mg/dL, first hour: 180 mg/dL, and second hour: 153 mg/dL) GDM is diagnosed. Carpenter -Coustan, NDDG, and O’Sullivan- Mahan criteria use a 3 h with 100 g glucose loading approach [26] with different cut-off values. GDM is confirmed when any two results exceed than thresholds [27, 28].

The sample size of the GDM study population varied among the studies, ranging from 7 to 305 participants.

The mean age of participants was 32.19 for GDM and 27.02 years for the control group and the mean pre-pregnancy BMI was 27.03 for GDM and 24.04 kg/m^2^ for the control group (mean of ages and BMI all studies are reported, except that two studies were conducted on obese women [11, 25]). Accordingly, women who developed GDM had higher BMI and were older than those who didn’t.

In 4 articles sampling had taken place in both second and third trimesters [11, 18, 20, 21], 3 articles in the second trimester [12–14], and 4 articles in the third trimester [15–17, 22]. There were also 3 articles with sampling in time of delivery [19, 23, 25], and one of the conference abstracts didn’t report the time at which sampling had been done [24].

Results have been reported in 2 categories: plasma free omega fatty acids (10 articles) [11–15, 20–22, 24, 25] and plasma phospholipid omega fatty acids (5 articles) [16–19, 23].

Nine studies applied GC-MS as an analytical technique [12–14, 16–18, 20–22] and others used NMR [11], LC-MS [24], FIA-MS/MS [15], or liquid-gas chromatography [19].

Four articles had estimated the intake of macronutrients and individual fatty acids during the pregnancy and evaluated the effect of dietary intake on plasma omega fatty acids [12, 16, 17, 20].

Data on population characteristics and metabolomic techniques in each study are summarized in Table 1 and the results of each study are summarized in Table 2.

**Table 1:** Characteristics of the Included Studies

	First auther/ year	Type of study	country	GDM/ control	age (year) GDM/ control	BMI (kg/m^2^) GDM/ control	Diagnostic criteria	sample type
1	White SL 2017	cohort	UK	198/ 448	31.5 ± 4.6 30.5 ± 5.6	36.1 (33.0–39.4) 34.7 (32.6–38.5)	IADPSG	plasma, serum or whole blood FFAs
2	Chen XH 2019	cohort	USA	81/ 1287	26.01 ± 6.05 21.90 ± 5.03	30.08 ± 6.42 25.37 ± 6.21	Carpenter/Coustan	plasma FFAs
3	Chen XH 2010	nested case-control	USA	49/ 98	25.63 ± 0.81 21.29 ± 0.54	30.76 ± 0.93 25.40 ± 0.56	Carpenter/Coustan	serum FFAs
4	Li XP 2020	nested case-control	China	305/ 305	30.6 ± 4.4 30.1 ± 4.1	22.4 ± 3.2 20.5 ± 2.6	IADPSG	plasma FFAs
5	Enquobahrie DA 2015	nested case-control	USA	178/ 180	34.0 ± 4.8 33.1 ± 4.3	26.7 ± 7.2 23.4 ± 5.3	Carpenter/Coustan	Serum FFAs
6	Lehmann R 2015	case-control	Germany	9/ 15	32 ± 1 30 ± 1	29.8 ± 2.5 25.6 ± 0.8	IADPSG	plasma FFAs
7	Burlina S 2016	case-control	Italy	21/ 21	33.9 ± 3.9 33.2 ± 4.6	24.6 ± 6.2 22.8 ± 4.0	Carpenter/Coustan	Plasma phospholipid fatty acids
8	Wijendran V 1999	case-control	Hartford, CT	15/ 15	32.13 ± 3.253 29.33 ± 3.99	25.95 ± 5.89 23.30 ± 2.98	O’Sullivan and Mahan	plasma phospholipid fatty acids
9	Zhu YY 2019	nested case-control	California	107/ 214	30.5 ± 5.7 30.4 ± 5.4	–	Carpenter/Coustan	plasma phospholipid fatty acids
10	Min YJ 2006	case-control	Korea	12/ 12	31.5 ± 4.4 28.3 ± 2.8	21.4 ± 2.6 21.0 ± 1.7	National Diabetes Data Group Criteria	plasma phospholipid fatty acids
11	Kumatani S 2019	cohort	Japan	17/ 188	–	–	–	plasma FFAs
12	Johansen NJ 2018	–	Denmark	9/ 6	31 ± 6 28 ± 3	31.6 ± 6.4 29.7 ± 5.4	–	plasma FFAs
13	De La Garza Puentes A 2016	Population based cohort	Spain	44/ 135	–	–	–	plasma phospholipid fatty acids
14	Muñoz-Nava, M. A. 2018	–	Mexico	50/ 50	–	–	–	serum FFAs
15	Poole A 2015	cohort	Galvestone, TX	7/ 6	33.6 ± 5.9 28.3 ± 5.2	41 ± 11.4 38 ± 6.2	–	serum FFAs

**Table 2:** Results of the included studies

	First author	sampling time (weeks)	w3 FAs	w6 FAs	w9/ w7 FAs	analytical technique
1	White SL	15–18	–	Decreased: LA	Increased: MUFA	NMR
		23–30	Increased: DHA	–	–	
2	Chen XH	16	Not Significant: ALA, EPA, DHA	Not Significant: LA, AA, DGLA	Not Significant: palmitoleic, oleic	GC-MS
3	Chen XH	15	Increased: EPA	–	Increased: palmitoleic	GC-MS
		20–28	Increased: ALA, EPA, DHA	Increased: LA, AA	Increased: palmitoleic, oleic, Total MUFAs	
4	Li XP	13	Increased: EPA, n-3/n-6 ratio Not Significant: ALA, DPA, DHA	Increased: GLA, DGLA Decreased: LA, EDA, AA/EPA ratio Not Significant: AA	Increased: hypogeic acid, oleic acid, Total MUFAs	GC-MS
5	Enquobahrie DA	16	–	Increased: LA	Increased: Oleic acid	GC-MS
6	Lehmann R	24–27	–	Decreased: AA	–	FIA-MS/MS
7	Burlina S	third trimester	Not Significant: ALA, EPA, DPA, DHA	Not Significant: LA, DGLA, AA	–	GC-MS
8	Wijendran V	third trimester	Increased: DHA Decreased: ALA, DPA, EPA	Increased: ratio of AA to DGLA Decreased: EDA, DGLA Not Significant: LA, AA	Decreased: oleic acid	GC-MS
9	Zhu YY	10–14	Decreased: DHA, Total n-3 PUFAs Not Significant: ALA, EPA, DPA	Increased: GLA, DGLA, Not Significant: LA, EDA, AA, DTA, n6-DPA	–	GC-MS
		15–26	Decreased: EPA, DPA, Total n-3 PUFAs Not Significant: ALA, DHA	Increased: DGLA Decreased: EDA, DTA Not Significant: LA, GLA, AA, n6-DPA	–	
10	Min YJ	delivery	Increased: DHA Decreased: ALA	Not Significant: LA, AA	Not Significant: Oleic, total MUFA	LC- GC
11	Kumatani S	12–23	–	Increased: LA	Increased: palmitoleic acid	GC-MS
		24–34	–	Decreased: LA	Decreased: palmitoleic acid	
12	Johansen NJ	third trimester	–	–	Decreased: oleic acid	GC-MS
13	De La Garza Puentes A	at delivery	Increased: EPA, DHA, n3 PUFAs, LC-n3 PUFAs	Increased: AA, LC-n6 PUFAs	–	–
14	Muñoz-Nava, M. A.	not reported	–	Decreased: LA	Increased: Oleic acid	LC-MS
15	Poole A	not reported	Decreased: ALA Not Significant: DHA	Decreased: LA Not Significant: AA	–	–

*Abbreviations*: *ALA* α-Linolenic acid, *EPA* Eicosapentaenoic acid, *DPA* Docosapentaenoic acid, *DHA* Docosahexaenoic acid, *LA* Linoleic acid, *GLA* Gamma-linolenic acid, *EDA* Eicosadienoic acid, *DGLA* Dihomo-gamma-linolenic acid, *AA* Arachidonic acid, *BMI* Body mass index, *FFA* Free fatty acid, *GC-MS* Gas chromatography-mass spectrometry, *NMR* Nuclear magnetic resonance, *LC-MS* Liquid chromatography-mass spectrometry, *FIA-MS* Flow injection analysis-mass spectrometry, *MUFA* Monounsaturated fatty acid, *PUFA* Polyunsaturated fatty acid, *LC-n3 PUFAs* Long-chain omega 3 polyunsaturated fatty acid, *LC-n6 PUFAs* Long-chain omega 6 polyunsaturated fatty acid

The level of DHA did not change significantly in the second trimester, but in most studies, the third trimester was associated with an increasing trend in this regard. The amount of eicosapentaenoic acid (EPA) measured during pregnancy was generally either increased or did not change significantly. Among the studies conducted in the second trimester, α-Linolenic acid (ALA) did not change significantly and its changes in the third trimester were very contradictory. Changes in omega-6 fatty acids during pregnancy were not significant, if significant, conflicting results were reported. Oleic acid and palmitoleic acid concentrations increased in the second trimester of pregnancy, but their changes in the third trimester were shown to be contradictory.

## Discussion

### Summary of evidence

This systematic review summarizes the available data on the association of circulating omega 3, 6, and 9 fatty acids with GDM. Most included studies have revealed correlations between metabolites and GDM risk factors, especially age, pre-pregnancy BMI, family history of T2D, smoking, ethnicity, systolic blood pressure, and parity. Just one of the included studies evaluated the frequency of physical activity during pregnancy. Studies mostly used Carpenter–Coustan criteria and then IADPSG criteria as diagnostic criteria. GC-MS was the most applied analytical technique.

Mammals can make saturated and omega-9 monounsaturated fatty acids but are unable to synthesize precursors required to produce omega-3 and omega-6 fatty acids which are needed for the body cells to function properly, so these fatty acids, called essential fatty acids, must be supplied through food. However, mammals can produce some longer-chain unsaturated fatty acids through unsaturation and elongation processes. Linoleic acid as an essential omega 6 fatty acid, is a precursor of dihomogamma linoleic acid, arachidonic acid (20:4 n-6, AA), and α-linoleic acid as an essential omega 3 fatty acid, is a precursor of EPA and docosaexahenoic acid (DHA) which are their long-chain metabolites (long-chain PUFAs, LC-PUFAs) [29]. AA and DHA are important components of the cell membranes. For instance, AA is used in the phospholipid part of the membrane of all cells and DHA is an important part of the phospholipids that make up the membrane of the central nervous system [30]. Also, prostaglandins and prostacyclins are among the eicosanoid metabolites of LC-PUFAs that play a key role in placental growth and development, gestational length, and initiation of labor [31].

Pregnancy is associated with changes in the mother’s metabolism to provide the fetus’ needs for proper growth. This period is associated with increased accumulation of lipids in maternal tissue and the later development of maternal hyperlipidemia [32]. During the first months of pregnancy, LC-PUFAs stored in the mother’s adipose tissue acts as the only source of LC-PUFAs for fetal growth [31].

### Dietary intake

Studies conducted on the connection between fatty acids received via dietary and their amounts (that is reflected in the blood) revealed that n-3 and n-6 PUFA, in comparison with saturated and monounsaturated fatty acids, have reliable diagnostic worthiness [10]. Omega-6 / omega-3 ratio is an important criterion for determining health. A balance in the consumption of omega 3 and 6 fatty acids is necessary to maintain health throughout the lifecycle as well as a successful pregnancy. An imbalance in this ratio leads to auto-immune and mental disorders, chronic inflammation, and diabetes. Over the past few decades, consumption of Western diets which consist of large amounts of omega-6 fatty acids has increased, leading to a loss of fatty acid balance [30]. A summary of national nutrition studies shows a reduction in the consumption of PUFA, especially omega-3 PUFA, derived from seafood on a global scale [10]. This issue is important and noteworthy in maintaining the health of people in the community, especially pregnant women who are carriers of the next generation.

### Insulin resistance

On the other hand, in pregnancy, to ensure the supply of nutrients needed for fetal growth, insulin resistance, and hyperinsulinemia can develop to some extent, which in some women leads to gestational diabetes [33]. The main pathophysiological causes of GDM are considered to be decreased insulin secretion and abnormal insulin resistance, which are associated with impaired fatty acid metabolism [30]. The amount of fat received through the diet and the type of fatty acids that make up the structure of these fats are among the factors that are effective in moderating insulin resistance.

Chen XH et al. reported when Homeostatic Model Assessment for Insulin Resistance (HOMA-IR) and C-peptide increased, GDM risk has increased by twofold to fourfold. There was a negative relationship between palmitoleic, oleic, linolenic acids, and HOMA-IR and C-peptide levels. In contrast, arachidonic, dihomo-γ-linolenic (DGLA), and DHAs were positively associated with HOMA-IR and C-peptide [12]. As reported by Muñoz-Nava MA et al., HOMA-IR positively correlated with dihomo-gamma linolenic acids, and negatively with oleic and linoleic acids. Beta-cell function in the GDM group positively correlated with linolenic acid and negatively with oleic acid [24]. In Zhu YY et al. study, among plasma phospholipid n-3 PUFAs at gestational weeks 10–14, EPA inversely correlated with insulin and HOMA-IR [18].

### Inflammatory markers

Diabetes is a metabolic disorder caused by increased Inflammation. Fatty acids in the diet are among the factors that can affect the inflammatory pathways [34]. Adipose tissue has a considerable role in GDM development through the synthesis and secretion of a great number of adipocytokines and biologically active materials such as proinflammatory cytokines, acute phase reactants, leptin, resistin, adiponectin, and others. These compounds can lead to insulin resistance when synthesized at high levels. There is a basic connection between fatty acids and inflammation, especially as precursors to eicosanoids as modifiers of inflammation [32]. Plasma total antioxidant capacity could be adjusted by the administration of omega-3 fatty acids and vitamin E in women with GDM [35].

In a study by Chen XH et al., cytokine or adipokine levels could be predicted by the individual FFAs. For instance, women with increased DGLA levels were twice as likely to have higher interleukin (IL)-8. Conversely, women who had higher oleic, palmitoleic, and linolenic acid, had decreased odds for having higher Interleukin-6 (IL-6), Interleukin-8 (IL-8), or tumor necrosis factor-alpha (TNF-α) [12]. Results of a study by Burlina S et al. show that all the inflammatory parameters considered (TNFa, IL6, IL-10, and C-reactive protein) were significantly higher in GDM women than in the NGT group, both during the pregnancy and after delivery [16]. In another study by Zhu YY et al., DHA correlated positively with adiponectin, among plasma phospholipid n-3 PUFAs at gestational weeks 10–14 [18].

### BMI

Pre-pregnancy BMI is a considerable risk factor of GDM by which the risks for developing GDM are 2–6 folds greater in women with either pre-pregnancy obesity or extensive gestational weight gain (GWG) [36]. In most obese individuals, elevated plasma FFA concentrations are observed because extended and stressed adipose cells release more FFA and FFA elimination may be diminished. Moreover, elevated FFA can prevent insulin’s antilipolytic action which leads to releasing more FFA into the blood circulation [37]. By characterizing the relationship between maternal BMI and GDM with maternal metabolites and neonatal or cord blood metabolites, the theory of the transgenerational cycle of obesity and diabetes can be verified [38].

In a study by White SL et al., it was indicated that obese GDM women exhibited overstated dyslipidaemic profiles compared with obese non-GDM women, which integrated the effects of insulin resistance in the lipid metabolism pathways and subsequently reduced insulin sensitivity from an earlier gestation in pregnant women [11]. De La Garza Puentes A et al. reported a BMI ≥ 25 in GDM women with a higher rate of LCn6 to LCn3 fatty acids [23]. Wijendran V et al. showed that in women with GDM, maternal plasma phospholipid DHA and n-3 long-chain PUFAs were significantly lower in overweight (BMI: > 25.5 to < 30) than in normal-weight (BMI > 19.8 to ≤25.5) subjects [17].

### Physiologic mechanisms of omega fatty acids

Omega-3 PUFA administration result in improvement of lipid profile, inflammatory markers, and glycemic state in GDM through PPAR-gamma which is the members of the nuclear receptors family that control various genes related to fatty acid metabolism [39]. The activity of PPAR-gamma in peripheral blood mononuclear cells (PBMCs) affected by Omega-3 PUFA, enhanced expression of LDL receptor, and decreased inflammatory markers. These accomplish were continued by reduction of fasting glucose, LDL, and triglycerides and rise of HDL levels. Regulation of lipid profile in GDM patients involved of diverse mechanisms. The normal transportation of fatty acids and their suitable concentration mediates by the placenta. The abnormal progress and function of the placenta underlie disturb of Omega-3 and Omega-6 PUFAs balance. Free fatty acid receptor 4 (FFAR4) is one of the factors that expresses in the placenta and has role in the insulin resistance mediation and adipocyte differentiation. The FFAR4 expression may be influenced by polymorphisms, so, they can influence the therapeutic consequences of Omega-3 PUFA administration. Supplementation with Omega-6 PUFAs indicated an increase in the AA levels in GDM which is associated with improved insulin sensitivity, glucose levels, and reducing obesity [40]. The beneficial effect of ω-3 and ω-6 FAs in GDM patients and their offspring have been reported while their role in GDM prevention is unclear. In some of the included studies the changes in levels of these fatty acids were associated with the risk of GDM with inconsistent results. Some others conducted metabolic profiling and it is not obvious the changes in fatty acids are the cause of GDM or these changes are the consequence of GDM. There is a need for cohort studies, especially cohorts in women from pre-pregnancy to postpartum to evaluate this issue.

## Conclusion

Women with GDM are at increased risk of developing complications of pregnancy. Therefore, prevention, timely diagnosis, proper management of GDM by controlling the amount of omega fatty acids intake is important to improve metabolic status, decrease inflammatory compounds, and long-term GDM-related complications. Introducing new methods for measuring the concentration and distribution of omega fatty acids in different stages of pregnancy is contributory in assessing gestational insulin resistance, BMI, and complementary therapy.

Among the evaluated fatty acids, ALA, DHA, EPA, oleic acid, and palmitoleic acid changed significantly in different trimesters and their changes in some other trimesters were contradictory. Changes in omega-6 fatty acids during pregnancy were not significant, if significant, conflicting results were reported.

There is a need for further investigation in regards to clinical consequences followed by employing these metabolites as predictors of GDM. Advanced findings regarding metabolite disturbances in GDM can be gained by similar investigations that evaluate the abundance of metabolites in more detail, incorporate metabolites with clinical characteristics, use multiple platforms, and analyses of various specimen kinds.

Although the amount of fats consumed through food was shown to be in correlation with the amount of free fatty acids circulating in the bloodstream, the impact of dietary intake in most of the studies was not assessed, or results presented conflicting relationships. Also, in studies that have measured the effects of diet on blood fatty acids, 24-h recalls have been used to assess the dietary behaviors. However, a 24-h assessment is not capable of showing a strong correlation between lipid intake and the blood level of fatty acid.

It can be one of the strengths of future studies, to design a large characterized study including the assessment of FFAs indices concurrent with insulin resistance or secretion, inflammatory biomarkers which have been accredited and accepted in experimental studies on a large scale. It would be possible to evaluate the distinction potential of the biomarkers by performing the analyses at an early stage of gestation which results in a reliable early diagnosis of GDM. Moreover, evaluation of the necessity of fasting before tests in pregnant women is yet another subject that can be investigated.

In this study, a part of the included studies were conference abstracts which just reported the significance of results without measuring the levels of omega fatty acids. Among original articles, some of them did not report the concentration levels, and some were reported as fold changes or adjusted results or expressed as a percent of total fatty acids whereas the measures of association were not consistently reported across studies. On the other hand, the amounts of omega fatty acids were measured in various structures such as free in plasma, plasma phospholipids, and erythrocyte phospholipids which didn’t have enough articles in each subtype, hence impeding meta-analysis.

This study is the first review to comprehensively and rigorously summarize the available data on the association between circulating omega 3, 6, and 9 fatty acids with GDM, and by increasing the number of articles in this field, we will repeat this review by performing a meta-analysis.

## Data Availability

All data generated or analyzed during this study are included in this published article.

## Abbreviations

GDM: Gestational diabetes mellitus
T2D: Type 2 diabetes
NGT: Normal glucose tolerance
IADPSG: International Association of Diabetes and Pregnancy Study Groups
HOMA-IR: Homeostatic Model Assessment for Insulin Resistance
ALA: α-Linolenic acid
EPA: Eicosapentaenoic acid
DPA: Docosapentaenoic acid
DHA: Docosahexaenoic acid
LA: Linoleic acid
GLA: Gamma-linolenic acid
EDA: Eicosadienoic acid
DGLA: Dihomo-gamma-linolenic acid
AA: Arachidonic acid
BMI: Body mass index
FFA: Free fatty acid
GC-MS: Gas chromatography-mass spectrometry
NMR: Nuclear magnetic resonance
LC-MS: Liquid chromatography-mass spectrometry
FIA-MS: Flow injection analysis-mass spectrometry
MUFA: Monounsaturated fatty acid
PUFA: Polyunsaturated fatty acid
LC PUFAs: Long-chain polyunsaturated fatty acid
IL-6: Interleukin-6
IL-8: Interleukin-8
TNF-α: Tumor necrosis factor-alpha
FFAR4: Free fatty acid receptor 4
PPAR: Peroxisome proliferator-activated receptor
PBMCs: Peripheral blood mononuclear cells
